# Neural activity mapping of bumble bee (*Bombus ignitus*) brains during foraging flight using immediate early genes

**DOI:** 10.1038/s41598-020-64701-1

**Published:** 2020-05-12

**Authors:** Shiori Iino, Yurika Shiota, Masakazu Nishimura, Shinichi Asada, Masato Ono, Takeo Kubo

**Affiliations:** 1 Department of Biological Sciences, Graduate School of Science, The University of Tokyo, Bunkyo-Ku, Tokyo, 113-0033 Japan; 2 Laboratory of Entomology, Graduate School of Agriculture, Tamagawa University, Machida-Shi, Tokyo, 194-8610 Japan; 3 Bioresource Sciences Major, Graduate School of Agriculture, Tamagawa University, Machida-Shi, Tokyo, 194-8610 Japan

**Keywords:** Molecular neuroscience, Animal behaviour

## Abstract

Honey bees and bumble bees belong to the same family (Apidae) and their workers exhibit a division of labor, but the style of division of labor differs between species. The molecular and neural bases of the species-specific social behaviors of Apidae workers have not been analyzed. Here, we focused on two immediate early genes, *hormone receptor 38* (*HR38*) and *early growth response gene-1* (*Egr1*), and late-upregulated *ecdysone receptor* (*EcR*), all of which are upregulated by foraging flight and expressed preferentially in the small-type Kenyon cells of the mushroom bodies (MBs) in the honey bee brain. Gene expression analyses in *Bombus ignitus* revealed that *HR38* and *Egr1*, but not *EcR*, exhibited an immediate early response during awakening from CO_2_ anesthesia. Both premature mRNA for *HR38* and mature mRNA for *Egr1* were induced during foraging flight, and mRNAs for *HR38* and *Egr1* were sparsely detected inside the whole MB calyces. In contrast, *EcR* expression was higher in forager brains than in nurse bees and was expressed preferentially in the small-type Kenyon cells inside the MBs. Our findings suggest that Kenyon cells are active during foraging flight and that the function of late-upregulated *EcR* in the brain is conserved among these Apidae species.

## Introduction

Eusocial bees, including honey bees and bumble bees, exhibit highly sophisticated sociality as represented by their caste differentiation and division of labor of workers. Their social behaviors, however, show some species-specific differences. The division of labor in honey bee workers is based on the age after eclosion^1,2^; older foragers convey information about the location of a food source with a unique dance called the “waggle dance” to communicate information to their nestmates about the location of a food source. On the other hand, the division of labor in bumble bees is based on their body size rather than age; smaller bumble bees remain in the hive to nurse their brood^3,4^ whereas larger workers are engaged in foraging outside the hive within a few days after eclosion^5^. In contrast to honey bees, bumble bee foragers do not communicate the location of their foraging success to their nestmates: successful foragers simply alert their nestmates to the presence of a food source by running about in the hive^6,7^. The molecular and neural mechanisms of honey bee foraging behavior are well investigated^8–12^, but few studies have examined bumble bee foraging behavior^13,14^.

The molecular and neural mechanisms underlying honey bee social behaviors have been investigated on the basis of the brain structure. The mushroom bodies (MBs), a higher-order center involved in learning and memory as well as in the integration of multimodal sensory information in the insect brain^15^, are implicated in foraging behavior in the European honey bee (*A. mellifera*)^16–18^. The MBs comprise intrinsic neurons termed Kenyon cells (KCs) that are classified into four subtypes: class I large (l)-, middle (m)-, small (s)-type KCs, and class II KCs, according to the size and position of their somata, and gene expression profiles^19–24^, which are conserved among Aculeate Hymenoptera^25^.

Previous studies used immediate early genes (IEGs), whose expression is rapidly upregulated after neuronal activation, to identify the brain regions related to certain behaviors^26^. Findings from these studies using a battery of IEGs, such as *kakusei* (noncoding RNA identified from *A. mellifera*)^27^*, Egr1* (*early growth response gene-1*, also known as *NGFI-A*, *Krox24*, *zif268*, and *zenk*)^28–31^, and *HR38* (*hormone receptor 38*, the subfamily of *nuclear receptor 4A*)^23,32–34^, suggested a possible role of the sKCs and some mKCs in the MBs in sensory processing during the foraging flight in honey bees^12^. In addition to *HR38*^35^, *EcR* (*ecdysone receptor*) and other ecdysone signaling genes, such as *DopEcR* (*dopamine/ecdysteroid receptor*), and *Ddc* (*dopa decarboxylase*) are also reported to be upregulated during the foraging flight in honey bees, raising the possibility that ecdysone signaling in the honey bee brain is involved in foraging behavior^36^.

In the present study, we analyzed two IEGs, *HR38* and *Egr1*, and late-upregulated *EcR* to evaluate neural activity in the bumble bee (*Bombus ignitus*, *Bi*) and honey bee (*Apis mellifera*, *Am*) during foraging flight to disclose common and species-specific features of the neural activity related to foraging. First, we confirmed that both *Bombus HR38* and *Egr1* exhibit an immediate early response similar to *Apis* IEGs. Next, we analyzed the expression pattern of three Bombus genes, *HR38*, *Egr1*, and *EcR*, in the forager brain under two experimental conditions. In the first condition, the hives were set in a greenhouse, which partly resembles the natural condition, and in the second condition, the hives were set in a laboratory flight-cage, enabling us to sample foragers according to the foraging time-course. We also performed *in situ* hybridization to detect the expression profiles of these genes in the forager brains. Our findings indicated that both premature mRNA for *HR38* and mature mRNA for *Egr1* were induced in the bumble bee brains during foraging flight and both mRNAs for *HR38* and *Egr1* were sparsely detected inside the whole MBs. On the other hand, we showed that expression of *EcR* in the brain was significantly higher in nurse bees than in foragers and expressed preferentially in the sKCs of the MBs in bumble bee foragers. These results suggest that neural activity in the forager brain and the function of ecdysone signaling in the sKCs are conserved among these two species.

## Materials and Methods

### Animals

**Figure 1:**
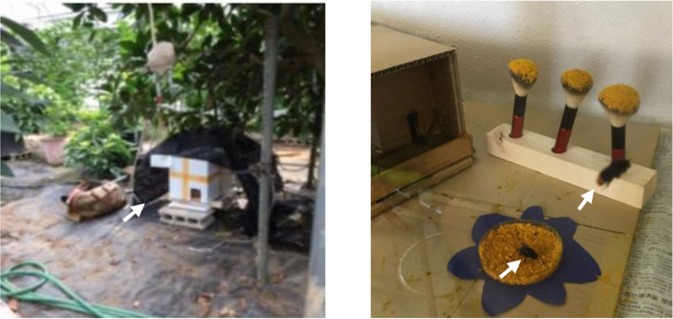
Experimental set-up used for *B. ignitus* worker sampling. (Left) *B. ignitus* colonies were set in a greenhouse. This photo shows a hive (white box in the center of the photo) set on a block and covered with a screen to shade the hive from direct sunlight in the greenhouse (arrow). (Right) A *B. ignitus* colony was set in a laboratory flight-cage. A hive with its entrance (wooden box) is on the left and a small dish supplied with pollen and three brushes attached with pollen as feeders are shown at the bottom and top of the photo, respectively. Note that one forager is collecting pollen at the feeder and another is flying to search for the feeder (arrowheads).

Bumble bee (*B. ignitus*) colonies at Tamagawa University (Machida-Shi, Tokyo, Japan) are usually kept under laboratory conditions (28 °C, 70% humidity, 24 h dark). For the present study, two colonies were placed in a greenhouse (Fig. 1, left panel) and three colonies were placed in a laboratory flight-cage (1 m × 50 cm × 50 cm, equipped with pollen feeding sites and absorbent cotton soaked in sugar water, Fig. 1, right panel) and maintained under laboratory conditions (25 ± 3 °C, 73 ± 5% humidity, and natural day/light hours) at Tamagawa University. A bumble bee colony was purchased from Agrisect Inc. (Inashiki-Shi, Ibaraki, Japan). Three European honey bee (*A. mellifera*) colonies were purchased from Kumagaya Apiary (Kumagaya-Shi, Saitama, Japan) and kept outside at the University of Tokyo (Bunkyo-Ku, Tokyo, Japan).

### Sampling for immediate early response analysis

A total of 70 *B. ignitus* workers were randomly collected from a colony and groups of 5 workers were divided into 14 insect cages (round plastic containers, 15 cm diameter and 4.5 cm high), and kept in a dark incubator at 25 °C overnight. The next morning at 8:00, all workers were set under a luminescent light in a laboratory space. Anesthesia was induced in 35 workers (7 insect cages) by supplying CO_2_ to the insect cages for seizure induction after hypoxia^37,38^, and 5 min later the CO_2_ in the 6 insect cages was exchanged with fresh air. The workers were collected at each time-point (0, 15, 30, 60, 120, and 180 min) after the CO_2_ was exchanged with fresh air (“CO_2_”). Workers anesthetized continuously with CO_2_ for 120 min were collected as a negative control (“NC”), in which we expected to detect no immediate early response. Another 35 workers (7 insect cages) that were supplied with air flow instead of CO_2_ were collected at the same time-points (10 min before the onset of the CO_2_ supply, 0, 15, 30, 60, 120, and 180 min after the CO_2_-fresh air exchange) as a series of positive controls (“PC”), in which we expected to detect the induction of IEGs due to the surrounding stimuli, but not due to exposure to high levels of CO_2_. After the bees were immediately anesthetized in iced water, the whole brains were dissected with fine tweezers and scalpels under a binocular microscope and then frozen at −80 °C for preservation.

### Sampling for foraging flight analysis

**Figure 2:**
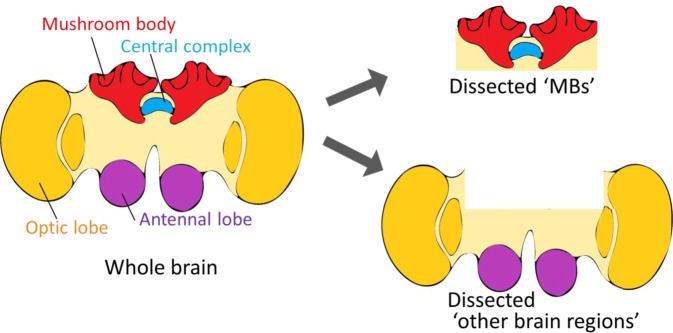
Brain dissection for qRT-PCR analysis. Schematic drawing of the brain dissection for qRT-PCR analysis. Dissected “MBs” mainly include the MBs and central complex. Dissected “other brain regions” mainly include the antennal lobes and optic lobes.

The sampling of *B. ignitus* foragers was performed in August 2018. For sampling in the greenhouse, “Foragers” that visited flowering fruit trees (*Pouteria lucuma*) with pollen loads and “Nurse bees” that were engaged in the in-hive tasks (feeding the brood, smoothing the nest combs, or warming eggs and pupae) were captured from the hives around 14:00. For sampling from the laboratory flight-cage, the day before the sampling day, all workers outside the hives were recovered in the hives and the hive entrances were closed. The next day at 8:30, the entrances were opened. The workers emerging from the entrances were immediately captured. At the same time, nurse bees in the hives were collected. Workers that were foraging around the pollen feeder at 3–7 min (8:37) and 25–30 min (9:00) after opening the entrance were collected. After they were anesthetized in ice water, the body size from the top of the head to the bottom of the abdomen of each bee was measured using a ruler with 1-mm resolution. For quantitative reverse transcription-polymerase chain reaction (qRT-PCR), the MBs and other brain regions were dissected as depicted in Fig. 2. Each dissected tissue was frozen at −80 °C for preservation.

Sampling of *A. mellifera* foragers was performed from September to October 2019. The day before the sampling day, the bees that returned to the hive with pollen loads were caught as foragers and marked on their thorax with a non-permanent marker pen. Early the next morning at 6:30, marked bees were collected from inside the hives, i.e., foragers that had not yet begun foraging that day. At 8:30, the hive entrances were opened and the workers exiting the hive were captured immediately. Foragers returned to their hives with pollen loads were collected at 9:00, 9:30, and 12:30, respectively. Nurse bees were also collected from inside the hives at 6:30, 9:30, and 12:30 based on their behaviors of plunging their heads into honeycomb cells that contained larvae more than twice as evidence of nursing their brood^39^. Half of the foragers and nurse bees captured at 12:30 were incubated in a dark incubator till 22:30. All the bees were promptly anesthetized in ice water and their brains were dissected for qRT-PCR as described above. Because the hypopharyngeal glands, which synthesize royal jelly, are well developed in nurse bees whereas they are shrunken in foragers^40^, nurse bees collected were further screened under a stereomicroscope to collect brains only from bees with well-developed glands^41^.

### qRT-PCR analysis

Expression analysis by qRT-PCR was performed essentially as described previously^31^ using TB Green *Premix Ex Taq II* (Tli RNaseH plus; Takara) and gene-specific primers (Table 1) with a Light Cycler 480 Instrument II (Roche Life Science, Indianapolis, IN, USA).

**Table 1:** Gene-specific primers.

Animal (analysis)	Gene name	NCBI Gene ID	Primer sequence	Size (bp)	Temp. (°C)
*A. mellifera* (qRT-PCR)	*HR38*	551592	5′-CGATTGGCTCCACAGTATTC-3′ and 5′-CTCCATGCGATGAGGCTCC-3′	136	58
	*preHR38*	551592	5′-TTATGTATGGACGTGCAGAC-3′ and 5′-ATCGGATACACGTCGATTAG-3′	125	52
	*EcR*	406084	5′-TACCACTACAACGCGCTCAC-3′ and 5′-CCTCATGTACATGTCGATCT-3′	120	56
	*Egr1*	726302	5′-CCTCACCACCCACGTGAGAA -3′ and 5′-TGCTTGAGGTGGACTTTGGC-3′	117	58
	*EF1α*	408385	5′-TTGTGCCGTGTTAATAGTCG-3′ and 5′-GATCGGTCATGTCCATCTTG-3′	149	56
	*Actin*	406122	5′-TCCCCGAATCCCGAAAG-3′ and 5′-CGGAGGAACCAAAGGACAA-3′	89	55
*B. ignitus* (qRT-PCR)	*HR38*	100642535	5′-CGATTGGCTCCACAGTATCC-3′ and 5′-CTCCATGCGATGAGGTTCC-3′	136	58
	*preHR38*	100642535	5′-TGACGAGCCTACGACATGTC-3′ and 5′-TGAATCGTGGAAGGCGAGTT-3′	139	58
	*EcR*	100646757	5′-TATCACTACAACGCACTGAC-3′ and 5′-CCGCATGTACATATCGATCT-3′	120	55
	*Egr1*	100651542	5′-CTTAACCACTCACGTGAGAA-3′ and 5′-TGTTTCAAGTGAACTTTCGC-3′	117	56
	*EF1α*	100631080	5′-TTGTGCCGTGTTAATAGTGG-3′ and 5′-GATCGGTCATGTCCATCTTG-3′	149	56
	*Actin*	100646910	5′-GTCTCGTTTCTCGACCATAG-3′ and 5′-ACTGATCTTCGAATGCCTAAA-3′	93	55
*B. ignitus* (*in situ* hybridzation)	*HR38*		5′-CAATCTTCTCACTACGTCCA-3′ and 5′-GGGATAGATAGTGCGCTTTC-3′	440	
	*EcR*		5′-CACTAATCAGCCCTCAGAAG-3′ and 5′-TCAAACTGAAGCACATCTCG-3′	566	
	*Egr1*		5′-GAATCTCCTGTCCCATCATC-3′ and 5′-TGTTTCAAGTGAACTTTCGC-3′	573	

NCBI, National Center for Biotechnology Information; Size, PCR product size; Temp., annealing temperature setting at Light Cycler.

The PCR conditions were as follows: 95 °C, 5 min, (95 °C, 10 s; annealing temperature of each gene is shown in Table 1, 10 s; 72 °C, 10 s) × 45 cycles, 65 °C, 1 min; 97 °C, 0 s; and 40 °C, 30 s. The selectivity of all primers was verified by agarose gel electrophoresis of the RT-PCR products amplified using *Ex Taq* Hot Start Version (Takara) and by analyzing melting curves of the qRT-PCR products (see Supplementary Fig. S1). The expression of each gene was normalized to that of *EF1α* and *Actin* (the expression level of each gene is shown in Supplementary Fig. S2). The relative expression was calculated using the ΔΔ Ct method (ABI user bulletin #20). The calibration samples were obtained at 0 min after the cessation of anesthesia in the CO_2_ group for the immediate early response validation of *B. ignitus* from the MBs of nurse bees in the greenhouse experiment of *B. ignitus*, from the MBs of nurse bees collected at 8:30 in the laboratory flight-cage experiment of *B. ignitus*, and from the MBs of nurse bees collected at 6:30 in the foraging flight experiment of *A. mellifera*.

### *In situ* hybridization analysis

We used 7, 2, and 2 forager brains collected for the above-described experiment in *B. ignitus* to evaluate *BiHR38*, *BiEcR*, and *BiEgr1* expression, respectively. Whole brains embedded in Tissue-Tek O.C.T. Compound (SAKURA Finetek) were frozen and sliced into 10-µm thick sections. The cDNA fragments, corresponding to the *BiHR38*, *BiEcR*, and *BiEgr1* coding regions, were amplified from *B. ignitus* cDNA using gene-specific primers (Table 1). *In situ* hybridization was performed with digoxigenin-labeled riboprobes essentially as described previously^42^. Images of the brain slices were obtained using an optical microscope (BX-50, Olympus) and multiple photos were merged using Adobe Photoshop (CS3 EXTENDED ver.10.0, Adobe Systems) if necessary.

### Statistical analysis

All statistics were performed using R statistical software (ver.3.3.3). For immediate early response analysis, two-way ANOVA (factor 1, treatment; factor 2, time) was performed. After that, for the CO_2_ and PC groups, one-way ANOVA, and Dunnett’s test (CO_2_ groups were compared with the 0 min group, the PC groups were compared with the −10 min group). To compare the NC group with the CO_2_ 0 min group, an F test followed by Student’s t test or Welch’s t test was performed. The expression of *EF1α* and *Actin* differed slightly among the groups (Tukey-Kramer test after two-way ANOVA, CO_2_-NC: p < 0.01, CO_2_-PC: p < 0.001, see Supplementary Fig. S2a). For the greenhouse sampling of *B. ignitus*, two-way ANOVA (factor 1, tissue; factor 2, bee type) was performed. After the F test, Student’s t test or Welch’s t test was performed to compare each tissue between bee type. The expression of *EF1α* and *Actin* did not differ significantly for each bee type, but did differ significantly for each tissue by two-way ANOVA (also see Supplementary Fig. S2b). For the laboratory flight-cage sampling of *B. ignitus*, three-way ANOVA (factor 1, tissue; factor 2, bee type; factor 3, time) was performed, and then the Tukey-Kramer test was performed to compare between bee type and flight time for each tissue. The expression of *EF1α* was significantly different depending on the tissue (p < 0.001) and bee type (p < 0.05), and the expression of *Actin* was significantly different depending on the bee type (p < 0.05) (also see Supplementary Fig. S2c). For the sampling of *A. mellifera*, three-way ANOVA (factor 1, tissue; factor 2, bee type; factor 3, time) was performed for all samples. For each tissue, the Tukey-Kramer test was used to compare the upregulation dependence on the time-course, and Student’s t test or Welch’s t test was performed after the F test to compare nurse bees and foragers at each time-point. The expression of *EF1α* was significantly different depending on the tissue (p < 0.001), bee type (p < 0.001), and time (p < 0.001), and the expression of *Actin* was significantly different depending on the bee type (p < 0.05) (also see Supplementary Fig. S2d). The body sizes of *B. ignitus* were compared using Student’s t test and the Tukey-Kramer test.

## Results

### Validation of *B. ignitus* immediate early response by qRT-PCR

We first examined whether *BiHR38*, *BiEgr1*, and *BiEcR* show an immediate early response. We also examined premature mRNA for *BiHR38* (termed as *preBiHR38*), because *HR38* is induced a little later than *Egr1*^43^. For this, *B. ignitus* workers were anesthetized with CO_2_ and seizures were induced by awakening them from anesthesia. After CO_2_ was supplied to the insect cages, all the bees fainted within 5 min. When the CO_2_ was exchanged with fresh air (cessation of anesthesia), some workers began to twitch their legs within a few minutes, which is a typical movement related to CO_2_-induced seizures^27^. Within 15 min, some bees got up and a few of them began to walk. Within 30 min, almost all workers were up, breathing with their abdomen, or grooming. Within 60 min, some workers were walking or flying, and approximately 60% of workers within 120 min and all of them within 180 min were actively walking or flying about the cage.

**Figure 3:**
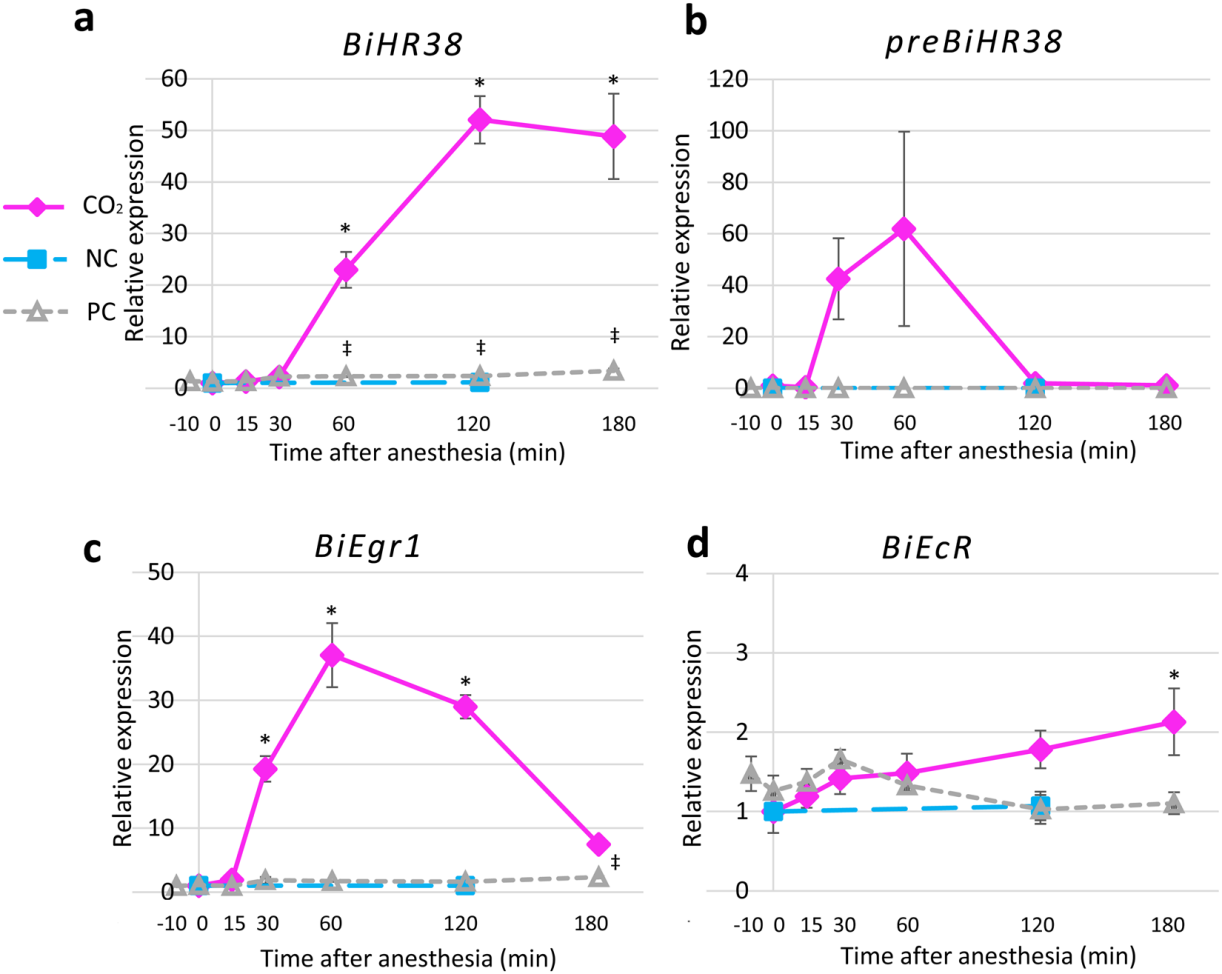
Analysis of *BiHR38*, *preBiHR38*, *BiEgr1*, and *BiEcR* expression levels after seizure induction. Time-course of the expression after awakening from CO_2_ anesthesia of *BiHR38* (**a**), *preBiHR38* (**b**), *BiEgr1* (**c**), and *BiEcR* (**d**). The expression level of each gene was analyzed by qRT-PCR and normalized with that of *BiEF1α*. Magenta lines indicate the group anesthetized with CO_2_ (“CO_2_”), light blue dashed lines indicate the negative control (“NC”, continuously anesthetized with CO_2_ for 120 min), and gray dotted lines indicate the positive control (“PC”, exposed just to air flow). All data indicate means ± SEM. Significant differences on the basis of Dunnett’s test after the ANOVA are indicated (*p < 0.05 for CO_2_ group, ^‡^p < 0.05 for PC group). Student’s t test and Welch’s t test revealed no significant difference between the NC group and the CO_2_ 0-min group. Some errors were so low that it is difficult to see the error bars in the graph. n = 5 for each sample.

The qRT-PCR results indicated that the *BiHR38* expression level normalized to that of *BiEF1α* changed depending on both the CO_2_ treatment and time after cessation of anesthesia (factor 1, 2: p < 0.001, two-way ANOVA). The relative *BiHR38* expression level increased beginning 30–60 min after the cessation of CO_2_ anesthesia, and peaked at 120 min (Fig. 3a). In contrast, in the NC group, which was continuously anesthetized by CO_2_ for 120 min, the *BiHR38* expression level did not change significantly compared with that at 0 min after the cessation of anesthesia as assessed by Student’s t test, indicating that the induction of *BiHR38* expression is associated with awakening from anesthesia. The *BiHR38* expression level in the PC group also changed slightly depending on the time after cessation of anesthesia. The *BiHR38* expression level in the CO_2_ group was 22-fold higher than that in the PC group at the upregulation peak at 120 min (mean relative expression [normalized by *EF1α*], CO_2_: 52.1, PC: 2.4). Essentially, the same results were obtained for the *BiHR38* expression level normalized with *BiActin* (Fig. S3a). The expression of *preBiHR38* in the CO_2_ group also tended to increase for 15–60 min, and then rapidly decreased at 120 min after the cessation of anesthesia (Fig. 3b). Although the expression did not differ significantly between the 15–60 min time points and that at 0 min (Fig. 3b), the *preBiHR38* relative expression was significantly different between 0 min and 60 min (Supplementary Fig. S3b; p < 0.05, Dunnett’s test). This finding suggests that *BiHR38* is an IEG induced in the brain by seizures. The expression of *BiEgr1* changed more rapidly than that of *BiHR38* (Fig. 3c and Supplementary Fig. S3c). The expression of *BiEgr1* increased beginning at 15–30 min, peaked at 60 min, and then decreased at 120 and 180 min. The expression changed depending on the treatment (factor 1: p < 0.001, two-way ANOVA) and time after cessation of anesthesia (factor 2: p < 0.001). The *BiEcR* expression level in the CO_2_ group differed significantly compared with that in the other two control groups (CO_2_-NC: p < 0.01, CO_2_-PC: p < 0.001, post hoc Tukey-Kramer test), as was also the case for *BiHR38*. In contrast, *BiEcR* expression increased only slightly (~2-fold) by 180 min after the cessation of anesthesia (mean of the relative expression in the CO_2_ group at 180 min compared with that at 0 min: 2.1; Fig. 3d and Supplementary Fig. S3d). The change in gene expression, however, was independent of both treatment and time after the cessation of anesthesia (factor 1: p = 0.16, factor 2: p = 0.42, two-way ANOVA). These findings indicated that both *BiHR38* and *BiEgr1*, but not *BiEcR*, exhibit an immediate early response.

### Analysis of gene expression of *B. ignitus* during foraging flight by qRT-PCR

**Figure 4:**
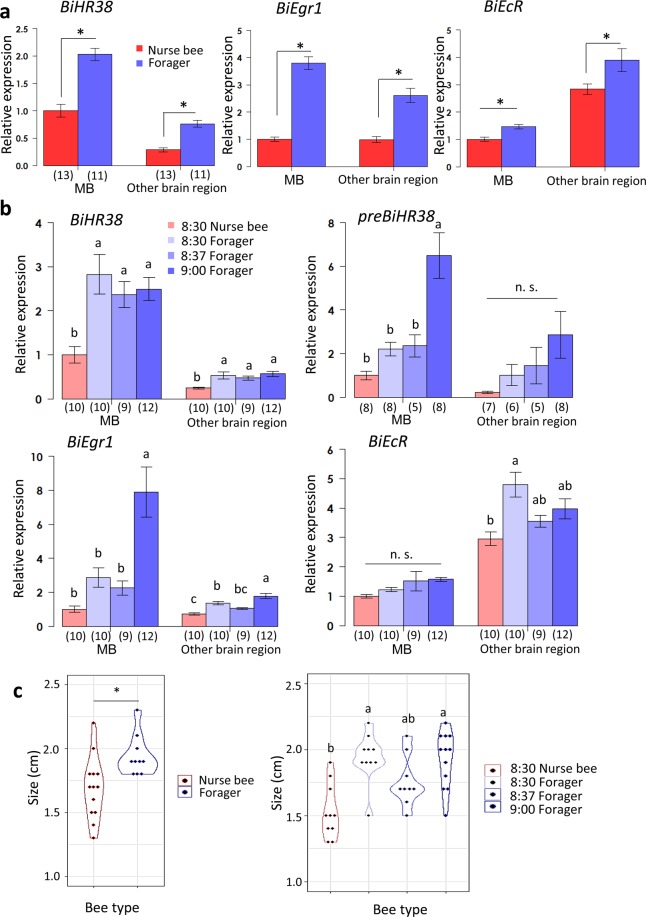
Analysis of *BiHR38*, *preBiHR38*, *BiEgr1*, and *BiEcR* expression during foraging flight by qRT-PCR. Expression analysis for the greenhouse experiment (**a**) and the laboratory flight-cage experiment (**b**). The expression level of each gene was analyzed by qRT-PCR and normalized with that of *BiEF1α*. Each bar represents the mean ± SEM. Significant differences are indicated by asterisks (p < 0.05, Student’s t test or Welch’s t test after the F test) on the error bars in (**a**), or different letters (p < 0.05, Tukey-Kramer test in (**b**), respectively). The sample size is indicated by the number in parentheses below the horizontal axis. (**c**) The body sizes of *B. ignitus* workers captured in the greenhouse (left) and those from the flight-cage (right). Significant differences are indicated by asterisks in the left panel (p < 0.05, Student’s t test), and different letters in the right panel (p < 0.05, Tukey-Kramer test) n.s., not significant.

First, we analyzed the expression levels of *BiHR38*, *BiEgr1*, and *BiEcR* of *B. ignitus* nurse bees and foragers captured in the greenhouse. We examined not only the MBs but also other brain regions to analyze the major brain regions that are active during the foraging flight. The gene expression level was normalized with that of either *BiEF1α* (Fig. 4a and Table 2) or *BiActin* (Supplementary Fig. S4a and Supplementary Table S1). The expression of *BiHR38* was significantly higher in foragers than in nurse bees in both the MBs (~2.0-fold) and the other brain regions (~2.7-fold; p < 0.05, Student’s t test in Fig. 4a, the expression folds were calculated from the mean of each group in Table 2). The expression of *BiEgr1*, which notably increases during the honey bee foraging flight^31,36,44^ was also significantly higher in foragers than in nurse bees in both MBs (~3.8-fold) and the other brain regions (~2.6-fold). The expression of *BiEcR* was also slightly but significantly higher in foragers than in nurse bees in both the MBs (~1.5-fold) and the other brain regions (~1.4-fold). Moreover, the expression levels of *BiHR38*, *BiEgr1*, and *BiEcR* differed significantly between the MBs and other brain regions (factor 1: p < 0.05, two-way ANOVA on each gene). Interestingly, however, whereas the expression of *BiHR38* and *BiEgr1* was higher in the MBs than in the other brain regions, *BiEcR* expression was higher in brain regions other than the MBs. Essentially, the same results were obtained when the gene expression level was normalized with *BiActin* (Supplementary Fig. S4a and Supplementary Table S1).

**Table 2:** Gene expression of *B. ignitus* in the greenhouse sampling normalized using *EF1α*.

Mean ± SEM	MB		Other brain region	
	Nurse bee	Forager	Nurse bee	Forager
*BiHR38*	1 ± 0.12	2.03 ± 0.11	0.28 ± 0.04	0.76 ± 0.06
*BiEgr1*	1 ± 0.08	3.80 ± 0.23	0.99 ± 0.10	2.62 ± 0.26
*BiEcR*	1 ± 0.08	1.47 ± 0.08	2.83 ± 0.19	3.89 ± 0.41

Relative gene expression in each brain tissue of workers normalized to *BiEF1α* in the *B. ignitus* greenhouse sampling compared with the MBs of nurse bees.

Next, we analyzed whether the expression levels of *BiHR38*, *BiEgr1*, and *BiEcR* in the MBs and other brain regions of foragers change during the foraging flight. *B. ignitus* foragers kept in the laboratory flight-cage exited the bee hive as soon as the hive entrance was open at 8:30. Because the feeder was so close to the hive entrance it took less than 30 min to complete a single foraging flight (i.e., searching for the feeder, obtaining pollen loads, and returning to the hive). The gene expression level was normalized with that of either *BiEF1α* (Fig. 4b and Table 3) or *BiActin* (Supplementary Fig. S4b and Supplementary Table S2). The expression level of *BiHR38* normalized with that of *BiEF1α* changed depending on the tissue and bee type, but did not change depending on the flight time (factor 1, 2: p < 0.001, factor 3: p = 0.57, three-way ANOVA)*. BiHR38* expression in both the MBs and other brain regions was significantly higher (~2.4–2.8-fold in the MBs and 1.9–2.3-fold in other brain regions) in foragers than in nurse bees at any time after the onset of foraging, while no significant change in *BiHR38* expression was detected in relation to the time after the onset of a foraging flight (p < 0.05, Tukey-Kramer [Fig. 4b], the expression folds were calculated from the mean of each group in Table 3). Significant upregulation of *preBiHR38* was observed in the MBs at 30 min after the onset of a foraging flight (p < 0.05, Tukey-Kramer test). The expression level of *BiEgr1* changed depending on the tissue and flight time, but did not change in relation to the bee type (factor 1, 3: p < 0.001, factor 2: p = 0.10, three-way ANOVA). *BiEgr1* expression in both the MBs and other brain regions was significantly higher in foragers at 30 min after the onset of foraging (~2.7-fold in the MBs and 1.3-fold in other brain regions comparing foragers at 8:30 and those at 9:00 in Table 3). Whereas, the expression level of *BiEcR* changed depending on the tissue and bee type (factor 1, 2: p < 0.001, factor 3: p = 0.22, three-way ANOVA), there was no significant upregulation in either tissue at any time-point after the onset of the foraging flight (Fig. 4b). Additionally, the *BiEcR* expression level in the MBs was higher (~1.6-fold) in foragers at 9:00 than in nurse bees, and that in the other brain regions was higher (~1.3-fold) in foragers at 9:00 than in nurse bees (Table 3). Essentially, the same results were obtained for gene expression levels normalized with that of *BiActin* (Supplementary Fig. S4b).

**Table 3:** Gene expression of *B. ignitus* in the laboratory flight-cage sampling normalized using *EF1α*.

Mean ± SEM	MB				Other brain region			
	8:30		8:37	9:00	8:30		8:37	9:00
	Nurse bee	Forager			Nurse bee	Forager		
*BiHR38*	1 ± 0.19	2.83 ± 0.45	2.37 ± 0.29	2.49 ± 0.26	0.25 ± 0.02	0.53 ± 0.08	0.48 ± 0.05	0.57 ± 0.06
*preBiHR38*	1 ± 0.20	2.21 ± 0.32	2.36 ± 0.51	6.48 ± 1.05	0.21 ± 0.05	1.02 ± 0.48	1.46 ± 0.83	2.85 ± 1.07
*BiEgr1*	1 ± 0.19	2.87 ± 0.56	2.25 ± 0.43	7.90 ± 1.46	0.73 ± 0.06	1.35 ± 0.10	1.05 ± 0.06	1.78 ± 0.14
*BiEcR*	1 ± 0.06	1.22 ± 0.07	1.51 ± 0.33	1.57 ± 0.07	2.95 ± 0.23	4.80 ± 0.42	3.54 ± 0.20	3.97 ± 0.34

Relative gene expression in each brain tissue of workers normalized to *BiEF1α* in the *B. ignitus* laboratory flight-cage sampling compared with the MBs of nurse bees collected at 8:30.

Taken together, these findings indicated that the expression of both *preBiHR38* and *BiEgr1*, but not *BiEcR*, significantly increased in association with the foraging flight (Fig. 4b). Moreover, the expression level of *BiHR38* and *BiEgr1* was higher in the MBs than in the other brain regions, whereas the expression level of *BiEcR* was higher in brain regions other than the MBs (Fig. 4a,b).

The body sizes of the foragers were significantly larger than those of nurse bees in both the greenhouse and laboratory experiments (Fig. 4c, p < 0.05, Student’s t test and the Tukey-Kramer test), which is consistent with a previous observation that relatively larger bumble bee workers tend to be engaged in foraging and smaller workers tend to engage in the in-hive tasks.

### Reexamination of gene expression in *A. mellifera* during foraging flight by qRT-PCR

**Figure 5:**
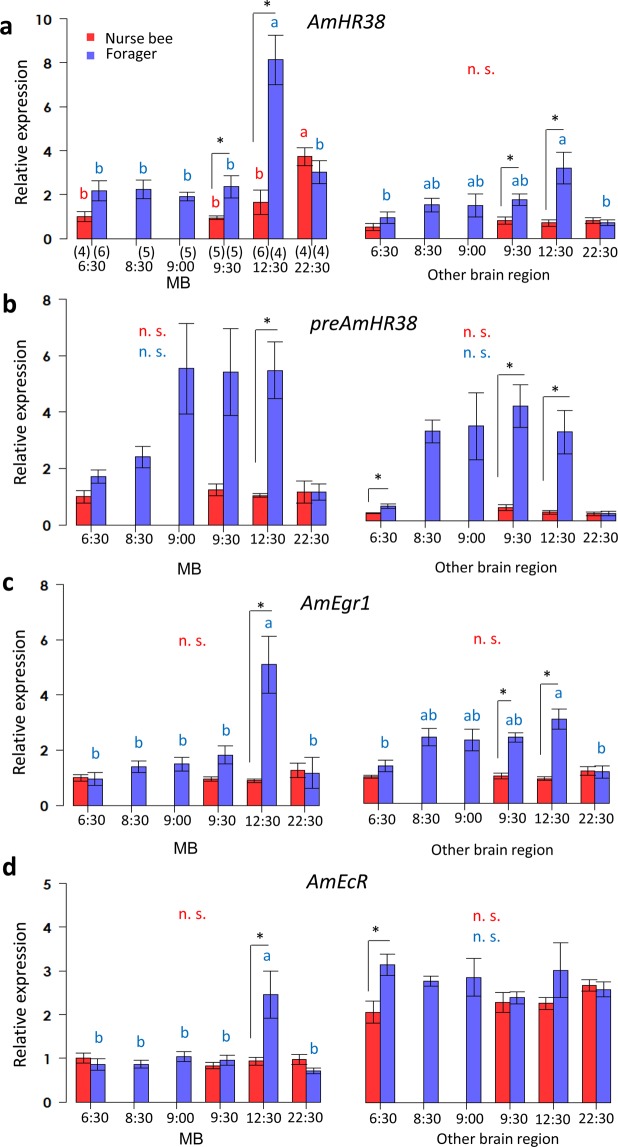
Analysis of *AmHR38*, *preAmHR38*, *AmEgr1*, and *AmEcR* expression during foraging flight by qRT-PCR. Expression levels of *AmHR38* (**a**), *preAmHR38* (**b**), *AmEgr1* (**c**), and *AmEcR* (**d**) were analyzed by qRT-PCR and normalized with that of *AmEF1α*. Each bar represents the mean ± SEM. Significant differences are indicated using different letters (p < 0.05, Tukey-Kramer test for nurse bees [red] and for foragers [blue] during the time-course, in each brain tissue respectively) or asterisks (p < 0.05, Student’s t test or Welch’s t test after the F test) on the error bars (black). The sample size is shown below the horizontal axis in parentheses. n.s., not significant.

To compare our results for *B. ignitus* with those for *A. mellifera*, we reexamined the neural activity of *A. mellifera* during foraging flight using the same sampling protocol. As seen in the laboratory flight-cage experiment for *B. ignitus*, *A. mellifera* workers came out from the bee hive as soon as the hive entrance was open at 8:30. The expression level of each gene was normalized with that of either *AmEF1α* (Fig. 5 and Table 4) or *AmActin* (Supplementary Fig. S5 and Supplementary Table S3). The relative expression levels of *AmHR38* normalized with that of *AmEF1α* changed depending on the tissue (the MBs or other brain regions), bee type (nurse bees or foragers), and time (factor 1, 2, 3: p < 0.001, three-way ANOVA). The expression level of *AmHR38* was not significantly different from that of nurse bees until 60 min after the onset of the foraging flight (9:30), and was significantly higher in active foragers at 12:30 than in nurse bees in both the MBs and other brain regions (~4.9-fold in the MBs and 4.5-fold in other brain regions, calculated with the data in Table 4). The expression decreased at 22:30 in foragers that were captured after the foraging flight and kept in a dark incubator for 10 h. Together, these findings indicate that *AmHR38* was induced by the foraging flight. *preAmHR38* was markedly upregulated from 30–60 min after the onset of the foraging flight and the expression level was maintained in foragers at 12:30 and again decreased in foragers at 22:30 (Fig. 5b). The change in *AmEgr1* expression also depended on the bee type and flight time (factor 2, 3: p < 0.01, factor 1: p = 0.61, three-way ANOVA). The expression level of *AmEgr1* at 12:30 was significantly higher in foragers than in nurse bees in both the MBs and other brain regions (Fig. 5c, ~5.7-fold in the MBs and 3.5-fold in other brain regions), and again decreased in foragers at 22:30. *AmEcR* expression in the MBs was also significantly higher (~2.6-fold) in foragers than in nurse bees at 12:30, but there was no significant difference in the *AmEcR* expression levels in the other brain regions between nurse bees and foragers at 12:30 (Fig. 5d, the same trend as shown in Supplementary Fig. S5d). Although gene expression levels in nurse bees also changed significantly at some sampling points (*AmHR38*, as seen in Fig. 5a at 22:30, *AmEgr1*, as seen in Supplementary Fig. S5c at 12:30 and 22:30, and *AmEcR*, as seen in Supplementary Fig. S5d between 6:30 and 12:30), significant differences were not consistently detected for the expression levels normalized with that of either *AmEF1α* or *AmActin*.

**Table 4:** Gene expression of *A. mellifera* normalized using *EF1α*.

Mean ± SEM	MB									
	6:30		8:30	9:00	9:30		12:30		22:30	
	Nurse bee	Forager	Forager	Forager	Nurse bee	Forager	Nurse bee	Forager	Nurse bee	Forager
*AmHR38*	1 ± 0.23	2.17 ± 0.47	2.25 ± 0.42	1.91 ± 0.20	0.95 ± 0.08	2.36 ± 0.50	1.65 ± 0.55	8.13 ±1.13	3.73 ± 0.40	3.01 ± 0.52
*preAmHR38*	1 ± 0.23	1.71 ± 0.24	2.40 ± 0.37	5.54 ± 1.60	1.25 ± 0.20	5.41 ± 1.54	1.04 ± 0.07	5.48 ± 1.01	1.15 ± 0.39	1.16 ± 0.30
*AmEgr1*	1 ± 0.12	0.98 ± 0.25	1.40 ± 0.22	1.50 ± 0.25	0.95 ± 0.07	1.82 ± 0.32	0.90 ± 0.06	5.11 ± 1.03	1.27 ± 0.26	1.17 ± 0.56
*AmEcR*	1 ± 0.11	0.86 ± 0.13	0.86 ± 0.09	1.04 ± 0.12	0.83 ± 0.07	0.96 ± 0.11	0.93 ± 0.09	2.46 ± 0.54	0.97 ± 0.12	0.71 ± 0.06
Mean ± SEM	Other brain region									
*AmHR38*	0.50 ± 0.16	0.92 ± 0.26	1.51 ± 0.31	1.50 ± 0.52	0.80 ± 0.16	1.75 ± 0.28	0.70 ± 0.15	3.17 ± 0.72	0.81 ± 0.13	0.70 ± 0.13
*preAmHR38*	0.26 ± 0.02	0.51 ± 0.07	3.17 ± 0.40	3.35 ± 1.18	0.45 ± 0.10	4.07 ± 0.76	0.29 ± 0.07	3.14 ± 0.78	0.24 ± 0.06	0.24 ± 0.08
*AmEgr1*	0.95 ± 0.06	1.34 ± 0.21	2.39 ± 0.32	2.29 ± 0.40	0.97 ± 0.11	2.38 ± 0.17	0.88 ± 0.06	3.04 ± 0.37	1.16 ± 0.15	1.13 ± 0.21
*AmEcR*	2.07 ± 0.26	3.15 ± 0.25	2.78 ± 0.11	2.87 ± 0.44	2.29 ± 0.23	2.40 ± 0.13	2.27 ± 0.14	3.03 ± 0.62	2.68 ± 0.13	2.60 ± 0.17

Relative gene expression in each brain tissue of workers normalized to *AmEF1α* in the *A. mellifera* sampling compared with the MBs of nurse bees collected at 6:30.

Taken together, these results suggested that both *HR38* and *Egr1* were significantly upregulated by foraging flight in both *B. ignitus* and *A. mellifera*, and a slight upregulation was also observed for late-induced gene *EcR*.

### Detection of activated cells by *in situ* hybridization

**Figure 6:**
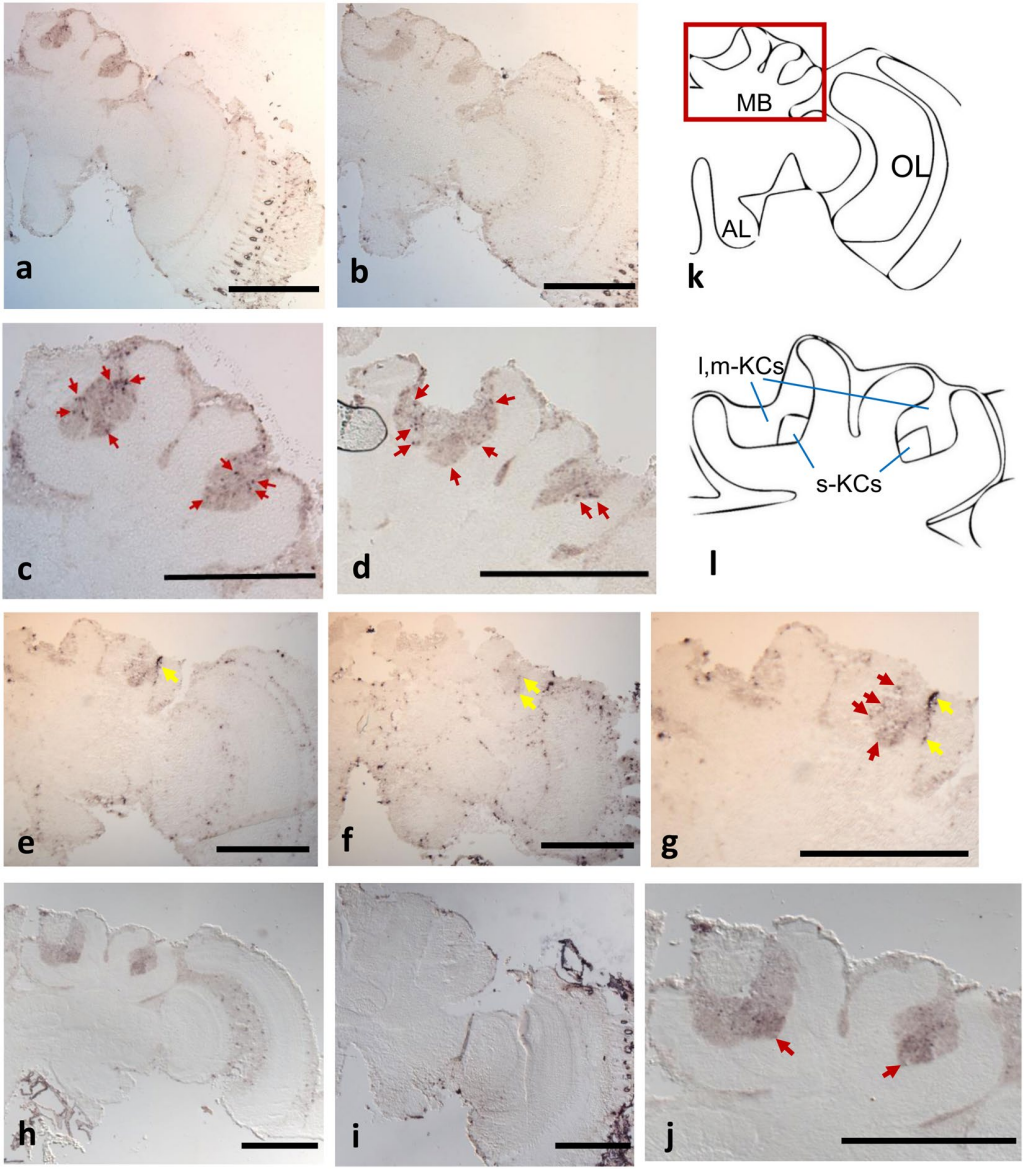
*In situ* hybridization of *BiHR38*, *BiEcR*, and *BiEgr1* in forager brain sections. Expression of *BiHR38* (a–d), *BiEgr1* (e–g), and *BiEcR* (h–j) was analyzed by *in situ* hybridization. Sections of brain hemispheres hybridized with antisense (a,e,h) or sense probes (b,f,i) are shown. Panel (k) indicates schematic drawing of the brain hemisphere and panel (l) indicates the magnified view of the MB enclosed by the red square in panel (k). Panels (c,d,g,j) indicate magnified views of the MB area in panels (a,e,h), respectively. (a–c) Forager collected at 9:00 in the laboratory flight-cage; (d–j) forager collected as “Forager” in the greenhouse. Representative signals are indicated by red arrows. Processing-induced damage to the tissue is indicated by yellow arrows (e–g, respectively). The s (m,l)-KCs: small-type (middle-, large-) Kenyon cells, AL: antennal lobe, OL: optic lobe. Scale bars = 500 µm.

We performed *in situ* hybridization analysis using brain sections of *B. ignitus* foragers to detect the cells activated in the forager brains. Both *BiHR38* and *BiEgr1* were strongly expressed in the MBs (Fig. 6a–d and e–g). Cells activated by *BiHR38* and *BiEgr1* were detected sparsely in the whole KCs (Fig. 6c,d,g). On the other hand, *BiEcR* was detected preferentially and locally in the small-type KCs in the MBs (Fig. 6h–j). *BiEcR* was also weakly detected in the whole brain cortex (Fig. 6h). Thus, consistent with our qRT-PCR results, these findings suggested that both *BiHR38* and *BiEgr1* were induced mainly in the MBs, and that *BiEcR* was expressed both in the sKCs and other brain regions, as previously reported in *A. mellifera*^23,31,45^.

## Discussion

After awakening from CO_2_ anesthesia, the expression of *preBiHR38*, *BiHR38*, and *BiEgr1* was prominently induced, indicating that both *HR38* and *Egr1* exhibit an immediate early response in the brains of *B. ignitus*. *BiEgr1* was induced earlier (~30 min after the cessation of anesthesia) than *BiHR38* (~60 min; Fig. 3a,c), which is consistent with previous studies in moths^34^, flies^34^, honey bees^31^, and mammals^43^. Expression of *BiHR38* and *BiEgr1* was very low, but significantly upregulated in the PC group, suggesting that novel surroundings, for example, light exposure or novel visual objects, stimulated the bees^44,46^. Expression of *preBiHR38* was also induced transiently within 30 min, earlier than *BiHR38* expression (Fig. 3a,b), which likely reflects the time needed for premature RNA to be processed into mature RNA. A previous study showed that induction of the expression of honey bee IEGs such as *kakusei* and *AmEgr1* is much more rapid – within 15–30 min after the cessation of anesthesia^27,31^. In contrast, our present results showed that the increased expression of both *preBiHR38* and *BiEgr1* was significant and prominent within 30 min after the cessation of CO_2_ anesthesia (Fig. 3a,c). These findings give rise to the possibility that CO_2_ treatment (~5 min), which was long enough to fully anesthetize the large *B. ignitus* workers, caused deep anesthesia and that it took a relatively long time for the workers to awake from the anesthesia, which resulted in a time-lag between the cessation of anesthesia and IEG expression. On the other hand, *BiEcR* expression was not significantly induced till 120 min (Fig. 3d), and was expected to be higher at 180 min after the awakening from anesthesia, indicating that *BiEcR* did not exhibit an immediate early response. Unexpectedly, expression of both *BiHR38* and *BiEgr1* was only slightly induced in bees treated with air instead of CO_2_ and exposed to the same luminescent light, which we originally expected to act as a positive control. It is possible that bees that had been kept in a dark incubator were exposed to some light and thus light exposure did not fully act as a stimulus to activate the IEGs.

In this experiment, we set colonies in a greenhouse or in a laboratory flight-cage for the sampling of *B. ignitus* workers, but we used colonies maintained outside for the sampling of *A. mellifera* workers. This was because honey bee workers tended to gather around the fluorescent lamp, making it difficult for them to forage normally in the restricted laboratory space. In contrast, the foraging of bumble bee workers did not seem to be affected in the greenhouse or in the laboratory flight-cage. In this small flight-cage, bumble bees workers tended to complete one foraging flight in less than 30 min, and therefore we collected workers at three time-points within 30 min after the onset of the foraging flight. We found that expression of *preBiHR38* and *BiEgr1* was upregulated 30 min after the onset of the foraging flight more prominently in the MBs than in the other brain regions, suggesting that the MB neural activity increased in the bumble bee during the foraging flight. It is plausible that induction of *preBiHR38* preceded that of *BiHR38* and eventually resulted in increased expression of *BiHR38* in foragers at 14:00. (Fig. 4a). In contrast, *BiEcR* was not significantly upregulated by the foraging flight at 30 min (Fig. 4b), which is consistent with our previous finding that *BiEcR* did not show an immediate early response until 120 min after awakening from anesthesia (Fig. 3d). The expression level of *BiEcR*, however, was also higher in foragers than in nurse bees collected at 14:00, like *BiHR38* and *BiEgr1* (Fig. 4a). This might be explained by the fact that the expression level of *BiEcR* was slightly, but significantly, higher in the CO_2_-treated group 180 min after awakening from anesthesia (Fig. 3d). It might be that *BiEcR* was upregulated later by the foraging experience, as reported previously^36^.

Although we first expected that *AmHR38*, *AmEgr1*, and *AmEcR* were upregulated in the brain within 30 min from the onset of foraging on the basis of a previous report, they were not upregulated even at the end of one foraging bout (Fig. 5a–d) except for *preAmHR38* (Fig. 5b). The expression of *AmHR38* did not change for 30 min after the onset of foraging when *preAmHR38* was already upregulated (Fig. 5b), suggesting that *AmHR38* could be induced by a single foraging flight longer than 30 min. This could be account for the finding that the *AmHR38* expression level was higher in foragers than in nurse bees collected at 12:30 (Fig. 5a). Also, neither *AmEgr1* nor *AmEcR* was upregulated in the MBs within 30 min after the onset of the foraging flight, but the expression of both was higher in foragers than in nurse bees collected at 12:30 and downregulated in foragers captured and kept in a dark incubator for 10 h (Fig. 5c,d). It might be that *AmEgr1* induction could not be detected 30 min after the onset of the foraging flight because we did not analyze the foraging flight time of individual workers: we might have collected workers that had been engaged in foraging for less than 30 min, even 30 min after the entrance was open to allow them to forage freely.

Also, in the present study, *BiHR38* and *BiEgr1* expression levels were high (~2-3-fold) in foragers at 8:30 before the onset of the foraging flight compared with that in nurse bees at the same time, and these genes were preferentially expressed in the MBs over the other brain regions (Fig. 4b). This was also at least partly true for the honey bees: the *AmHR38* expression level was higher in foragers than in nurse bees at 6:30 (~2-fold; Fig. 5a). We assume that the MB neural activity of foragers at that time was already upregulated, although the foragers inside the hive had not yet engaged in foraging. Eusocial bee foragers (*A. mellifera* and *B. terrestris*) have a steady circadian rhythm as they work outside in daylight and are influenced by temperature, whereas the circadian rhythm in nurse bees is attenuated as they work all day in a dark hive with a constant temperature^10,47,48^. Bumble bee (*B. terrestris*) foragers in the wild become active at almost 6:00 when they are turning out from the hive by ones and twos^49^. In our study, the hive entrance was shut until 8:30, which is the usual start time for bumble bees to forage. We assume that the foragers recalled the start time for foraging and their foraging-related neural activity was induced, as previously reported^36,50^. *Egr1* is suggested to be an IEG that is upregulated before the onset of foraging by the reward learning associated with time^51^. Social bee foragers must deal with changes in the good flowering locations according to the time of day. At the end of the day, the foragers must remember the best feeding locations at the different times to be ready for the next day. Thus, for effective foraging, the induction of *HR38* in the MBs – the higher center for memory and learning – in the early morning may reflect neural activity needed to recall their foraging experience.

In the *B. ignitus* brain, both *BiHR38* and *BiEgr1* were expressed sparsely in the entire MBs of the foragers captured at 14:00 in the greenhouse, contrary to a previous report that both genes were expressed preferentially in the sKCs in the brains of honey bee foragers^23,31^ (Fig. 6d,g). It is thus possible that the differences in the expression patterns in the MBs of *HR38* and *Egr1* are related to the species-specific traits of the honey bee and bumble bee foraging behaviors. It is also possible that they only reflect differences in the experimental conditions set for the honey bees and bumble bees (e.g., flight distance, flight speed, or feeder). It is necessary to discriminate these two possibilities in future studies.

It is noteworthy that *EcR* was expressed preferentially in the sKCs in the MBs in both *B. ignitus* and *A. mellifera* (Fig. 6j). Ecdysone signaling is suggested to be involved not only in molting or metamorphosis, but also in various social behaviors in insects^52^. Although three types of class I KCs in the MBs are reported to be conserved among Aculeate insects^25^, EcR protein is distributed in the whole MBs in *Camponotus japonicus*^53^. It might be that the functions of EcR in the brain differ between Formicidae and Apoidea, and are conserved among two Apidae species: the bumble bees and honey bees.

In conclusion, our findings suggested that the brain neural activity evoked by foraging flight are at least partly conserved among two social bee species; honey bees and bumble bees. Especially, it is possible that the functions of late-upregulated *EcR* in the sKCs is conserved among these two species. Further studies focusing on the signaling cascade that involves these IEGs are needed to confirm the above hypothesis.

## Supplementary information

Supplemental information.
