# Supplementary material for: Neural activity mapping of bumble bee (Bombus ignitus) brains during foraging flight using immediate early genes
Source: Sci Rep. 2020 May 12;10:7887. doi: 10.1038/s41598-020-64701-1 (PMC7217898; doi:10.1038/s41598-020-64701-1)
Supplement: Supplementary file 1 — Supplemental information. [file 41598_2020_64701_MOESM1_ESM.pdf]

Neural activity mapping of bumble bee (*Bombus ignitus*) brains during foraging flight using immediate early genes

Shiori Iino[1], Yurika Shiota[1], Masakazu Nishimura[2], Shinichi Asada[3]*, Masato Ono[2]* and Takeo Kubo[1]*

[1] Department of Biological Sciences, Graduate School of Science, The University of Tokyo, Bunkyo-Ku, Tokyo, 113-0033, Japan.
[2] Laboratory of Entomology, Graduate School of Agriculture, Tamagawa University, Machida-Shi, Tokyo, 194-8610, Japan.
[3] Bioresource Sciences Major, Graduate School of Agriculture, Tamagawa University, Machida-Shi, Tokyo, 194-8610, Japan.
Correspondence and requests for materials should be addressed to T.K. (email: stkubo@bs.s.u-tokyo.ac.jp)

*: Corresponding authors

Supplementary table S1

Mean ± SEM	MB		Other brain region	
	Nurse bee	Forager	Nurse bee	Forager
BiHR38	1±0.22	1.86±0.13	0.13±0.02	0.31±0.02
BiEgr1	1±0.14	3.70±0.31	0.48±0.07	1.09±0.07
BiEcR	1±0.10	1.44±0.11	1.39±0.10	1.67±0.09

Supplementary Table S1. The gene expression of *B. ignitus* in the greenhouse sampling normalized using *Actin*

Relative gene expression in each brain tissue of workers normalized to *BiActin* in the *B. ignitus* greenhouse sampling compared with the MBs of nurse bees.

Supplementary table S2

Mean ± SEM	MB				Other brain region			
	8:30		8:37	9:00	8:30		8:37	9:00
	Nurse bee	Forager			Nurse bee	Forager		
BiHR38	1±0.16	2.38±0.40	2.00±0.31	2.47±0.80	0.16±0.01	0.22±0.05	0.19±0.02	0.30±0.03
preBiHR38	1±0.14	1.82±0.28	2.03±0.54	6.44±1.54	0.20±0.04	0.49±0.19	0.65±0.34	1.76±0.67
BiEgr1	1±0.15	2.30±0.48	1.94±0.44	9.76±3.38	0.65±0.06	0.70±0.10	0.53±0.05	1.10±0.12
BiEcR	1±0.09	1.03±0.10	1.14±0.10	1.29±0.23	1.55±0.14	1.83±0.12	1.41±0.13	1.72±0.08

Supplementary Table S2. The gene expression of *B. ignitus* in the laboratory flight-cage sampling normalized using *Actin*

Relative gene expression in each brain tissue of workers normalized to *BiActin* in the *B. ignitus* laboratory flight-cage sampling compared with the MBs of nurse bees collected at 8:30.

Supplementary table S3

Mean ± SEM	MB									
	6:30		8:30	9:00	9:30		12:30		22:30	
	Nurse bee	Forager	Forager	Forager	Nurse bee	Forager	Nurse bee	Forager	Nurse bee	Forager
AmHR38	1±0.06	2.31±0.48	3.15±1.11	4.71±2.55	0.91±0.10	2.74±0.49	2.28±0.93	11.79 ±2.01	2.75±0.33	2.93±0.41
preAmHR 38	1±0.10	1.93±0.43	3.14±0.60	15.17 ±9.01	1.17±0.21	6.26±1.71	1.32±0.15	7.88±1.74	0.76±0.17	1.08±0.21
AmEgr1	1±0.29	0.86±0.21	1.54±0.36	3.46±2.12	0.76±0.06	1.79±0.31	0.96±0.09	6.18±1.53	0.76±0.14	0.90±0.37
AmEcR	1±0.30	0.75±0.11	1.00±0.27	1.84±0.81	0.65±0.05	0.94±0.10	0.99±0.13	2.82±0.51	0.59±0.08	0.58±0.05

Mean ± SEM	Other brain region									
	6:30		8:30	9:00	9:30		12:30		22:30	
	Nurse bee	Forager	Forager	Forager	Nurse bee	Forager	Nurse bee	Forager	Nurse bee	Forager
AmHR38	0.17±0.06	0.36±0.13	0.54±0.12	0.53±0.15	0.24±0.04	0.57±0.08	0.43±0.10	1.39±0.33	0.45±0.05	0.23±0.00
preAmHR 38	0.09±0.01	0.18±0.03	1.07±0.05	1.14±0.35	0.14±0.03	1.34±0.25	0.18±0.05	1.34±0.35	0.13±0.03	0.07±0.01
AmEgr1	0.27±0.04	0.42±0.10	0.72±0.12	0.70±0.08	0.26±0.02	0.66±0.05	0.46±0.04	1.10±0.15	0.55±0.06	0.31±0.03
AmEcR	0.55±0.06	0.96±0.18	0.83±0.09	0.88±0.10	0.65±0.13	0.66±0.02	1.17±0.08	1.09±0.23	1.43±0.42	0.75±0.10

Supplementary Table S3. The gene expression of *A. mellifera* normalized using *Actin*
Relative gene expression in each brain tissue of workers normalized with *AmActin* in the *A. mellifera* sampling compared with the MBs of nurse bees collected at 6:30.

Supplementary Figure S1

a

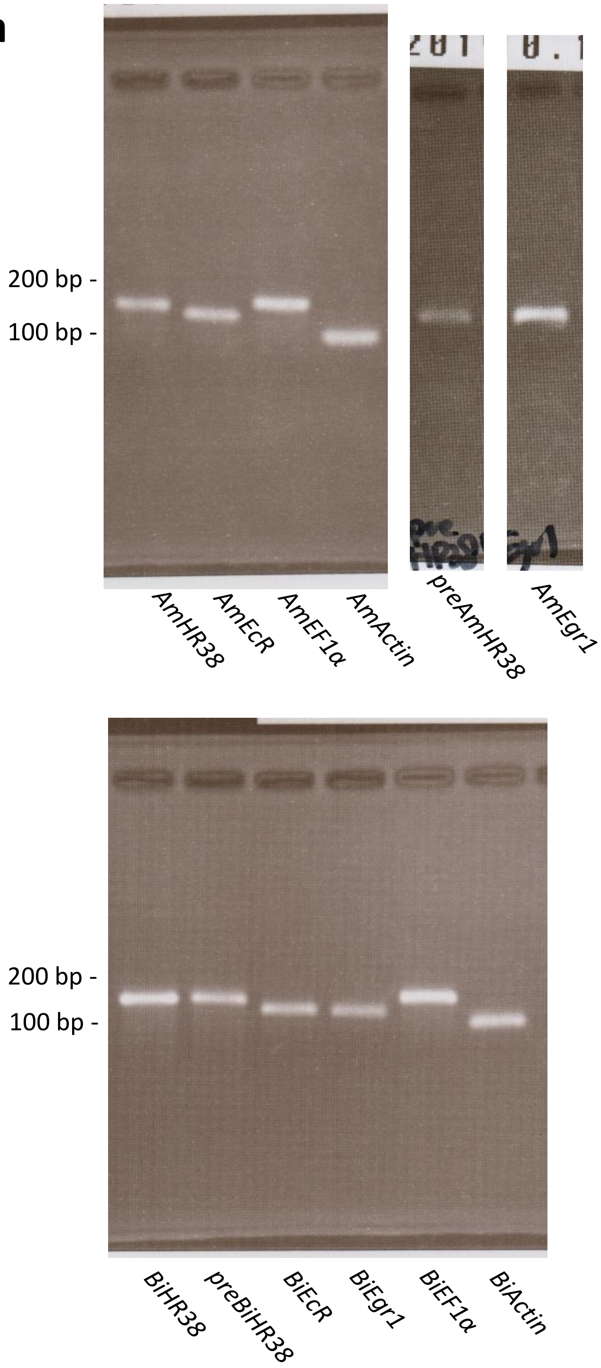

b

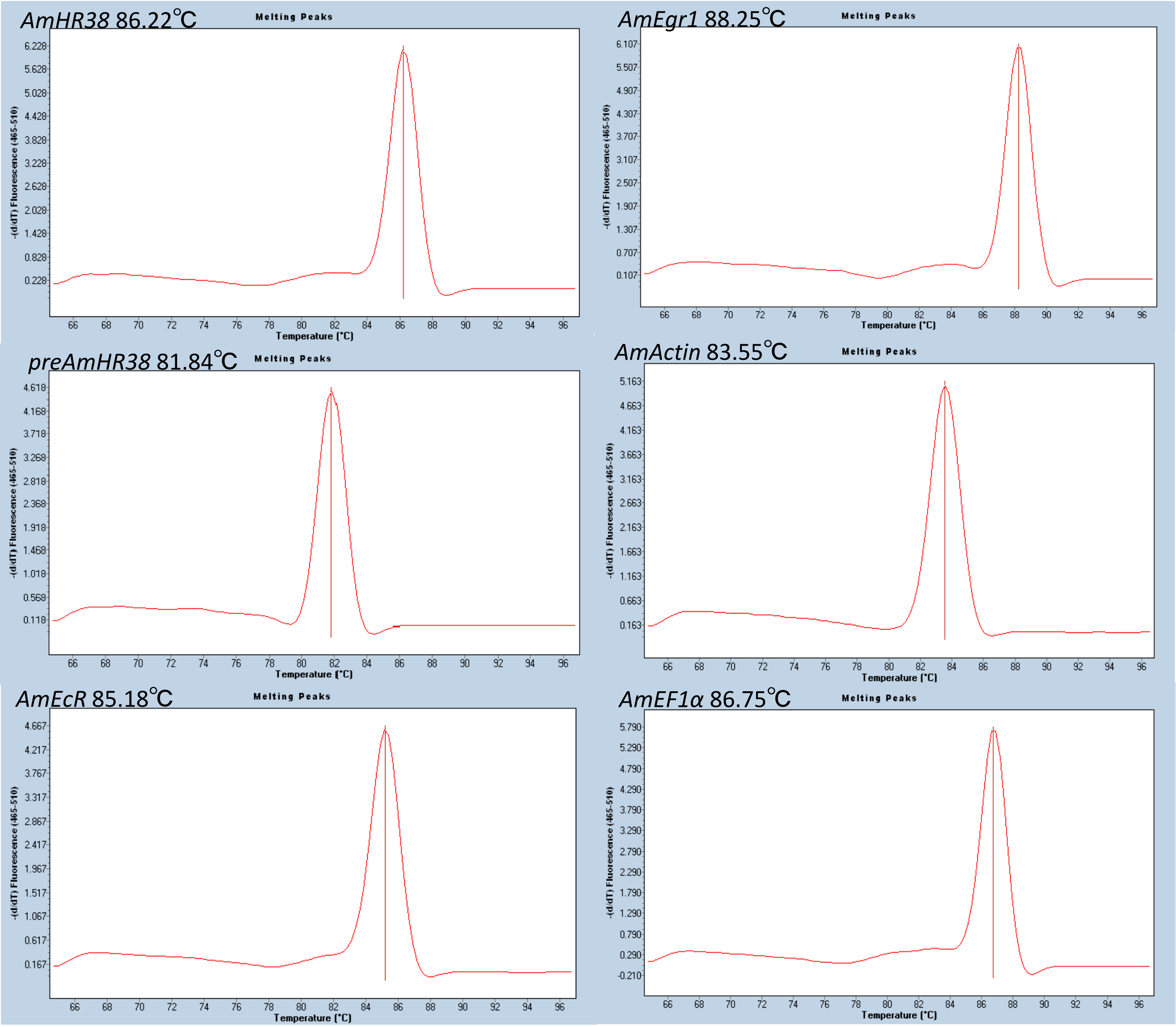

AmHR38 86.22°C
Melting Peaks
-(d/dT) Fluorescence (465-510)
Temperature (°C)
AmEgr1 88.25°C
Melting Peaks
-(d/dT) Fluorescence (465-510)
Temperature (°C)
preAmHR38 81.84°C
Melting Peaks
-(d/dT) Fluorescence (465-510)
Temperature (°C)
AmActin 83.55°C
Melting Peaks
-(d/dT) Fluorescence (465-510)
Temperature (°C)
AmEcR 85.18°C
Melting Peaks
-(d/dT) Fluorescence (465-510)
Temperature (°C)
AmEF1α 86.75°C
Melting Peaks
-(d/dT) Fluorescence (465-510)
Temperature (°C)

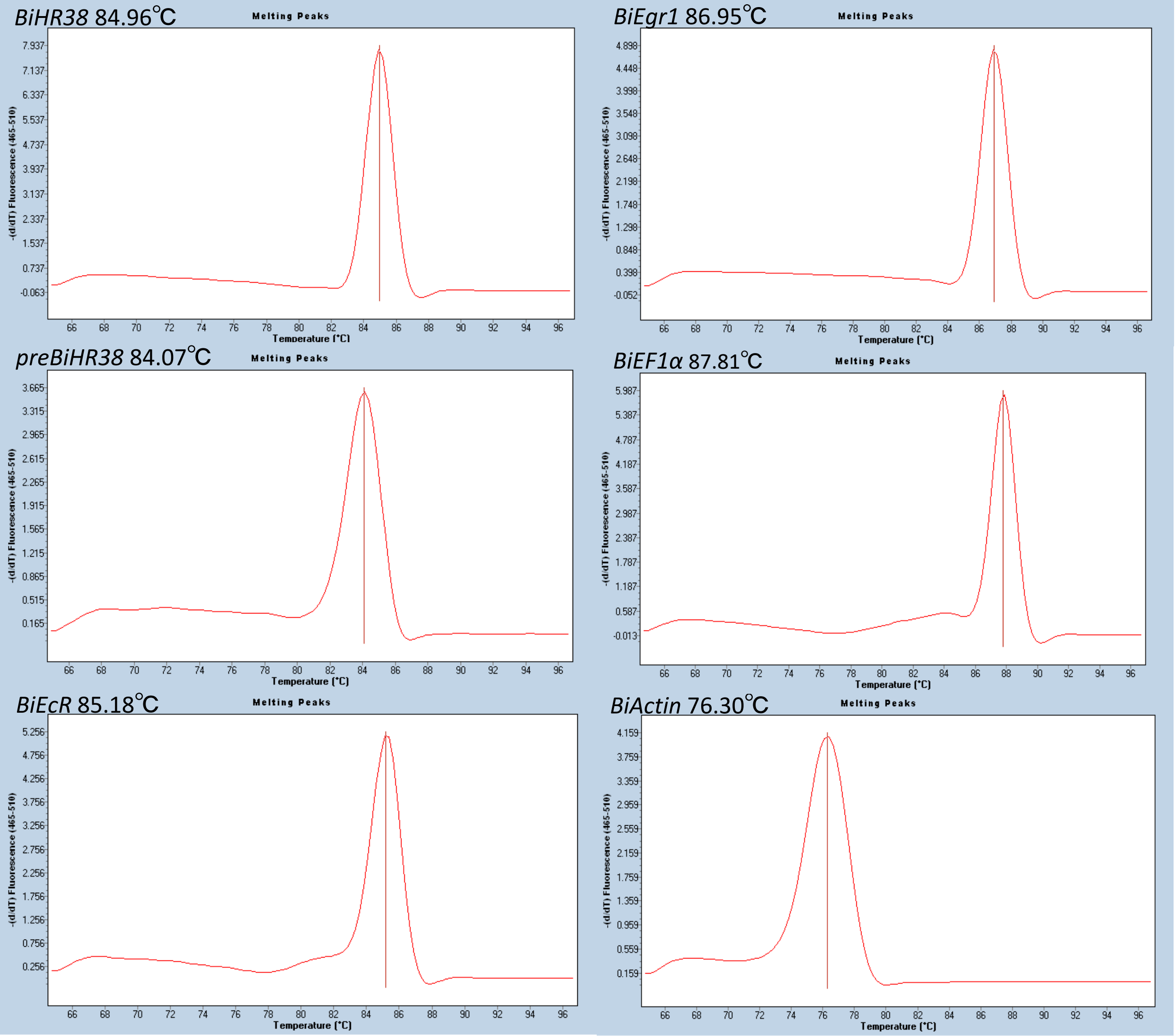

Supplementary Figure S1. Validation selectivity of gene-specific primers

(a) Agarose gel electrophoresis of each transcript of RT-PCR using gene-specific primers. The upper panel shows RT-PCR transcript using *A. mellifera* cDNA, and the lower panel shows that using *B. ignitus* cDNA. Full-length gel images were cropped from different gels and grouped with white spaces. (b) Melting peaks of qRT-PCR.

Supplementary Figure S2

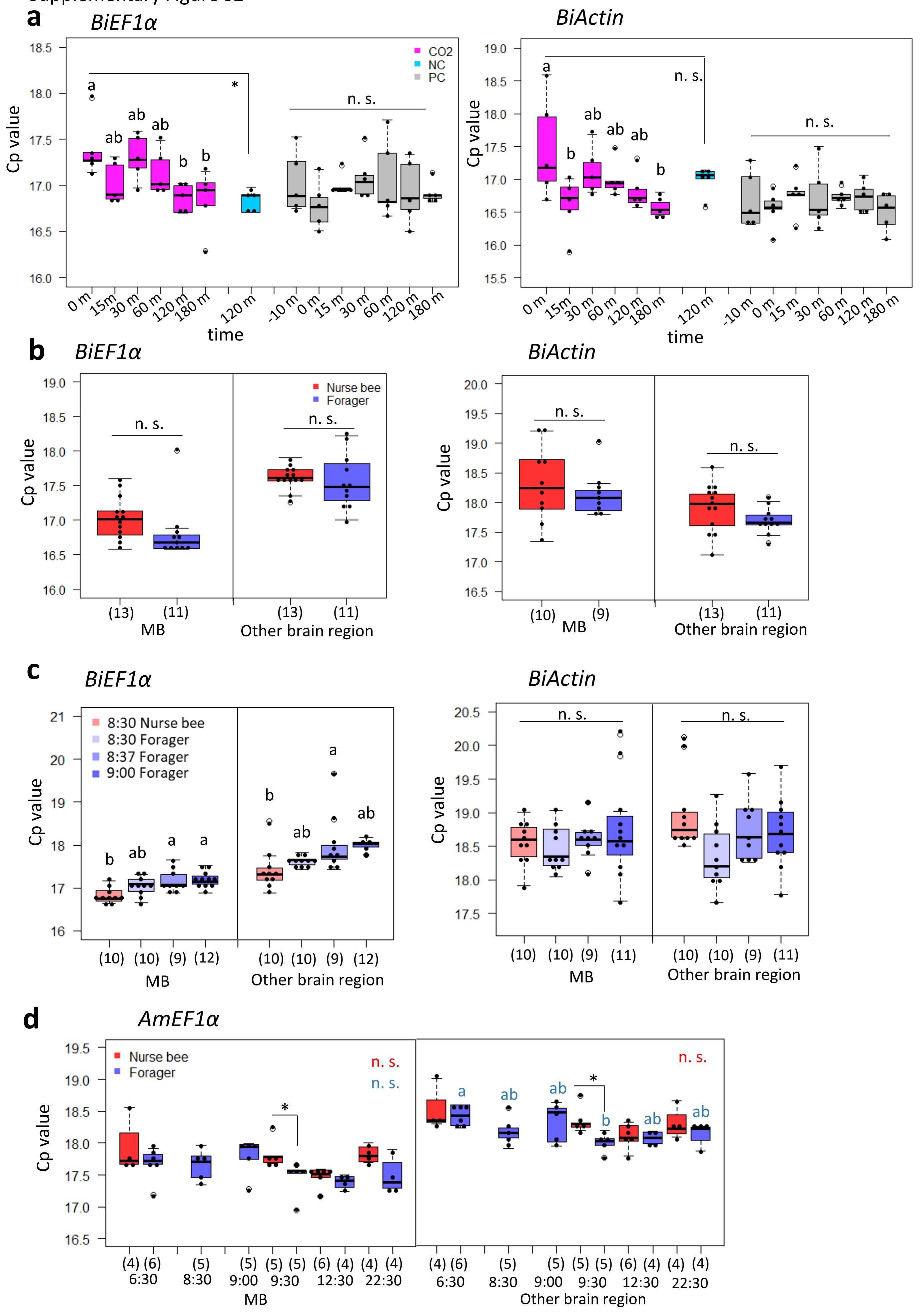

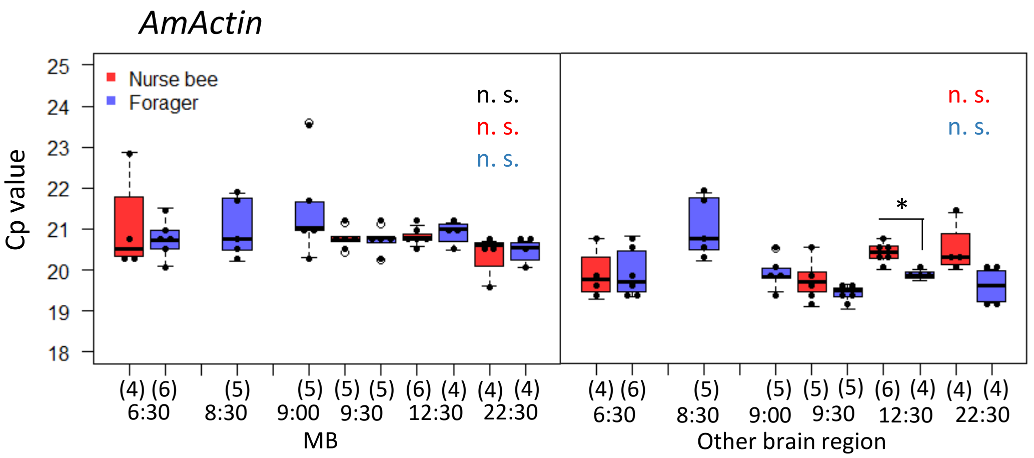

Supplementary Figure S2. The expression levels of *EF1α* and *Actin*

Time-course of the expression after awakening from CO_2 anesthesia (a). Magenta indicates the group anesthetized with CO_2 (“CO_2”), light blue indicates the negative control (“NC”, continuously anesthetized with CO_2 for 120 min), and gray indicates the positive control (“PC”, exposed to just an air flow). Significant differences are indicated using different letters among experimental groups ($p<0.05$, Tukey-Kramer test) or asterisks between “CO_2” 0 min and “NC” 120 min ($p<0.05$, Student’s t test or Welch’s t test after the F test). n=5 for each sample. (b) The greenhouse experiment for *B. ignitus*. Significant differences are indicated with asterisks for each tissue ($p<0.05$, Student’s t test or Welch’s t test after the F test). (c) The flight-cage experiment for *B. ignitus*. Significant differences are indicated using different letters for each brain tissue ($p<0.05$, Tukey-Kramer test). (d) The experiment for *A. mellifera*. Significant differences are indicated using different letters for nurse bees (red) and foragers (blue) during the time-course ($p<0.05$, Tukey-Kramer test), and asterisks (black) ($p<0.05$, Student’s t test or Welch’s t test after the F test at each time-point) for each tissue. The sample size is indicated by the number in parentheses below the horizontal axis. n. s., not significant.

Supplementary Figure S3

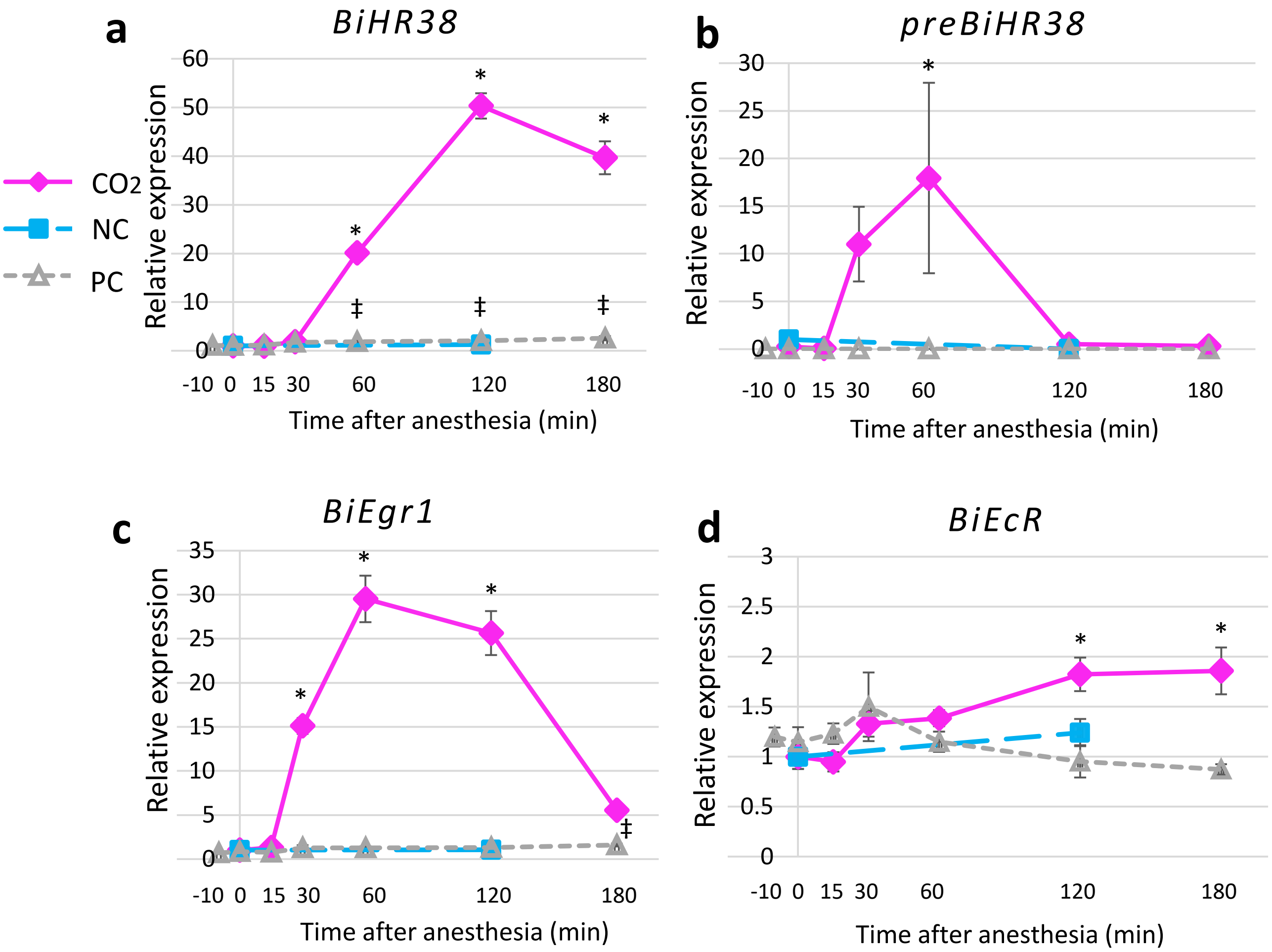

Supplementary Figure S3. Analysis of *BiHR38*, *preBiHR38*, *BiEgr1*, and *BiEcR* expression levels after seizure induction

Time-course of the expression of *BiHR38* (a), *preBiHR38* (b), *BiEgr1*(c), and *BiEcR* (d) after awakening from CO_2 anesthesia . The expression level of each gene was analyzed by qRT-PCR and normalized using *BiActin*. Magenta lines indicate the group anesthetized with CO_2 ("CO_2"), light blue dashed lines indicate the negative control ("NC", continuously anesthetized with CO_2 for 120 min), and gray dotted lines indicate the positive control ("PC", exposed to an air flow). All data indicate means ± SEM. Significant differences are indicated using Dunnett's test after ANOVA (*:$p<0.05$ for CO_2 group, ‡: $p<0.05$ for PC group). Student's t test and Welch's t test revealed no significant difference between the NC group and CO_2 0-min group. n=5 for each sample.

Supplementary Figure S4

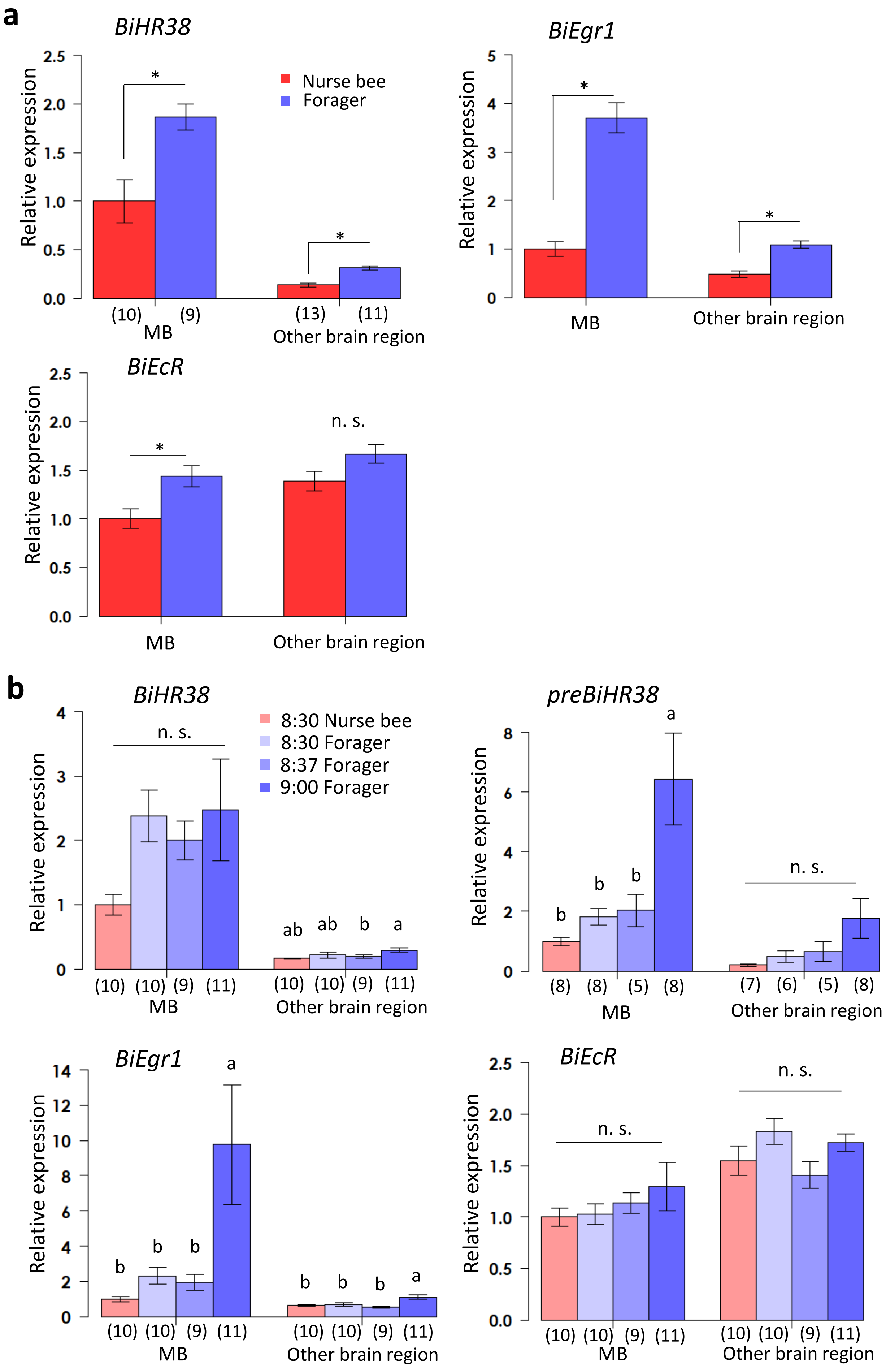

Supplementary Figure S4. Analysis of *BiHR38, preBiHR38, BiEgr1*, and *BiEcR* expression levels during foraging flight.

Expression analysis for the greenhouse experiment (a) and the laboratory experiment (b). The expression level of each gene was analyzed by qRT-PCR and normalized with that of *BiActin*. Each bar represents the mean ± SEM. Significant differences are indicated by asterisks ($p<0.05$, Student's t test or Welch's t test after the F test) on the error bars in (a), or different letters ($p<0.05$, Tukey-Kramer test in (b), respectively). The sample size is shown below the horizontal axis in parentheses. n.s., not significant.

Supplementary Figure S5

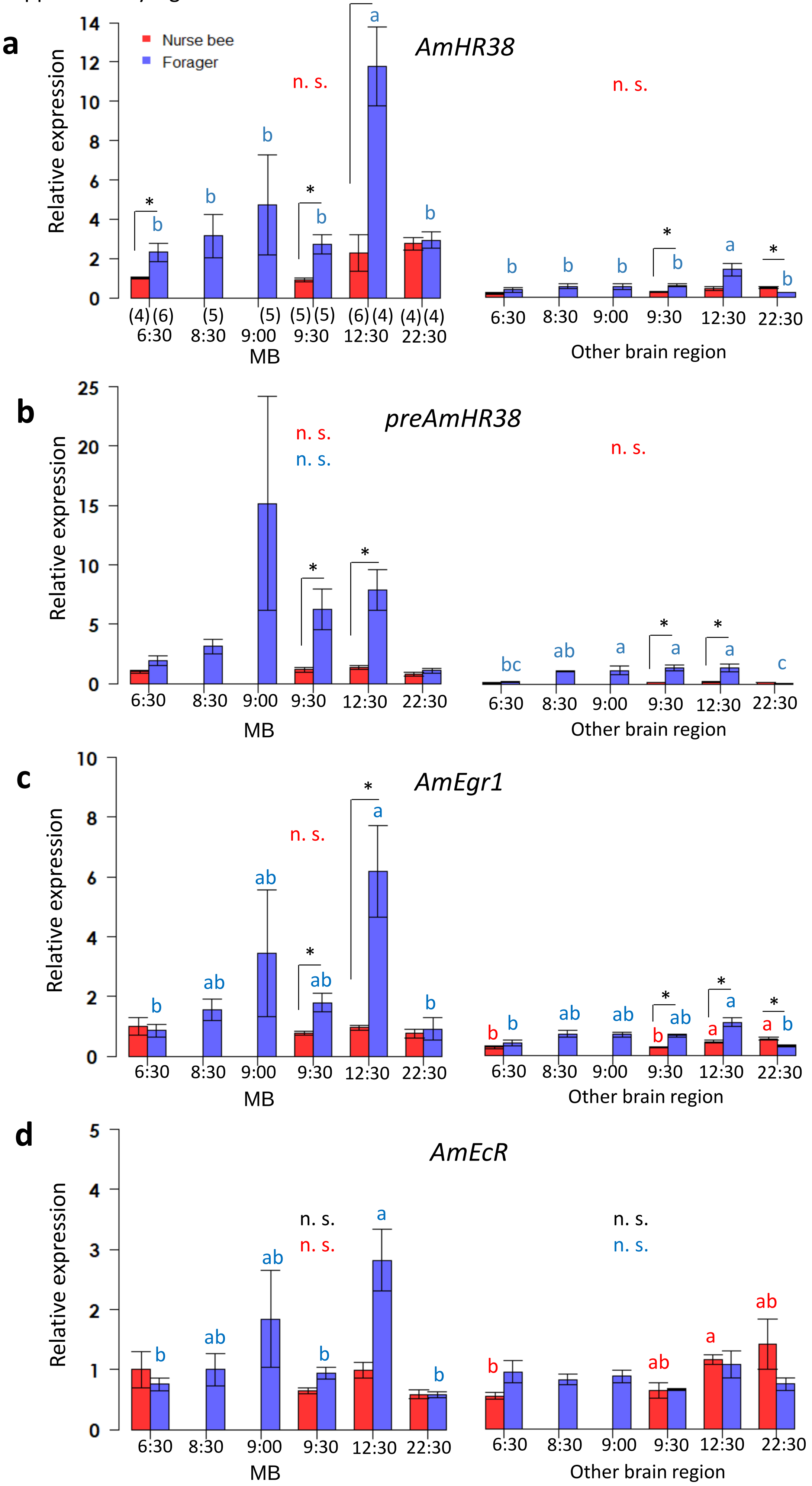

Supplementary Figure S5 Analysis of *AmHR38*, *AmpreHR38*, *AmEgr1*, and *AmEcR* expression levels during foraging flight

Expression levels of *AmHR38* (a), *preAmHR38*(b), *AmEgr1*(c) and *AmEcR* (d) were analyzed by qRT-PCR and normalized with that of *AmActin*. Each bar represents the mean ± SEM. Significant differences are indicated using different letters ($p<0.05$, Tukey-Kramer test for each bee type during the time-course, in each brain tissue, respectively) or asterisks ($p<0.05$, Student's t test or Welch's t test after the F test) on the error bars. The sample size is shown below the horizontal axis in parentheses. Some bars are too small to see in the *preAmHR38* graph. n.s., not significant.
